# Relationship of polymorphisms in microRNAs -124 e -126 with diabetic retinopathy in patients with type 2 diabetes

**DOI:** 10.1186/1758-5996-7-S1-A214

**Published:** 2015-11-11

**Authors:** Evelise Regina Polina, Renan C Sbruzzi, Maria Enoia Dantas C Silva, Luis H Canani, Daisy Crispim, Kátia Gonçalves dos Santos

**Affiliations:** 1 Universidade Luterana do Brazil, Porto Alegre, Brazil

## Background

MicroRNAs (miRs) are small non-coding molecules that regulate gene expression post-transcriptionally by promoting translational repression or degradation of target mRNAs. Altered expression as well as mutations in the sequences of miRs or their target sites have been related to a great number of diseases, including diabetes and its complications. The G allele of the rs531564 (C>G) polymorphism in miR-124 gene was already associated with increased risk of type 2 diabetes mellitus (T2D) and the A allele of the rs4636297 (G>A) polymorphism of miR-126 gene was associated with severity of diabetic retinopathy (DR).

## Objective

To investigate the association of rs531564 (C>G) polymorphism of miR-124 gene and rs4636297 (G>A) polymorphism of miR-126 gene with the presence and severity of DR in patients with T2D.

## Materials and methods

The sample comprises 627 T2D patients, including 248 subjects without DR, 223 with nonproliferative DR and 156 with proliferative DR. In addition, a population sample of 139 healthy blood donors was also analyzed. Patients were enrolled at four public hospitals in the State of Rio Grande do Sul and blood donors were recruited at one public hemotherapy center. Genotyping for both polymorphisms was done by real-time polymerase chain reaction method. Results: Genotype frequencies were in agreement with those expected by the Hardy-Weinberg equilibrium. Genotype and allele frequencies of rs531564 (miR-124) and rs4636297 (miR-126) polymorphisms in T2D patients and blood donors were not statistically different. The minor allele frequency (C allele) of rs531564 (miR-124) was 12% in T2D patients and 14% in blood donors (p=0.308); and the minor allele frequency (A allele) of rs4636297 (miR-126) was 43% and 38%, respectively (p=0.149). The evaluation of genotype and allele frequencies of the polymorphisms in miR-124 and miR-126 genes with the presence and severity of DR did not show significant differences. The minor allele frequency of miR-124 was 10% in patients without DR, 13% in nonproliferative DR and 14% in proliferative DR (p=0.349); and the minor allele frequency of miR-126 was quite similar in the three groups of T2D patients, approximately 43% (p=0.925).

## Conclusions

We did not find an association of two polymorphisms in miR-124 and miR-126 genes with the presence and severity of DR in patients with type 2 diabetes. However, further studies are needed to understand the role of polymorphisms in miRs genes in DR.

